# First Evidence of Inbreeding, Relatedness and Chaotic Genetic Patchiness in the Holoplanktonic Jellyfish *Pelagia noctiluca* (Scyphozoa, Cnidaria)

**DOI:** 10.1371/journal.pone.0099647

**Published:** 2014-06-30

**Authors:** Giorgio Aglieri, Chiara Papetti, Lorenzo Zane, Giacomo Milisenda, Ferdinando Boero, Stefano Piraino

**Affiliations:** 1 Dipartimento di Scienze e Tecnologie Biologiche e Ambientali (DiSTeBA), Università del Salento, Lecce, Italy; 2 Consorzio Nazionale Interuniversitario per le Scienze del Mare (CoNISMa), Roma, Italy; 3 Dipartimento di Biologia, Università di Padova, Padova, Italy; 4 Alfred Wegener Institute, Helmholtz Centre for Polar and Marine Research, Bremerhaven, Germany; 5 Italian National Research Council - Institute for Marine Science (CNR-ISMAR), Genova, Italy; Tuscia University, Italy

## Abstract

Genetic drift and non-random mating seldom influence species with large breeding populations and high dispersal potential, characterized by unstructured gene pool and panmixia at a scale lower than the minimum dispersal range of individuals. In the present study, a set of nine microsatellite markers was developed and used to investigate the spatio-temporal genetic patterns of the holoplanktonic jellyfish *Pelagia noctiluca* (Scyphozoa) in the Southern Tyrrhenian Sea. Homozygote excess was detected at eight loci, and individuals exhibited intra-population relatedness higher than expected by chance in at least three samples. This result was supported by the presence of siblings in at least 5 out 8 samples, 4 of which contained full-sib in addition to half-sib dyads. Having tested and ruled out alternative explanations as null alleles, our results suggest the influence of reproductive and behavioural features in shaping the genetic structure of *P. noctiluca*, as outcomes of population genetics analyses pointed out. Indeed, the genetic differentiation among populations was globally small but highlighted: a) a spatial genetic patchiness uncorrelated with distance between sampling locations, and b) a significant genetic heterogeneity between samples collected in the same locations in different years. Therefore, despite its extreme dispersal potential, *P. noctiluca* does not maintain a single homogenous population, but rather these jellyfish appear to have intra-bloom localized recruitment and/or individual cohesiveness, whereby siblings more likely swarm together as a single group and remain close after spawning events. These findings provide the first evidence of family structures and consequent genetic patchiness in a species with highly dispersive potential throughout its whole life cycle, contributing to understanding the patterns of dispersal and connectivity in marine environments.

## Introduction

Short life cycles, conferring the ability to mobilize huge populations that rapidly appear and disappear, allow gelatinous zooplankton to swiftly colonize available ecological spaces [1]. In particular, scyphozoans often form massive outbreaks that influence both ecosystem functioning and human activities (reviewed in [2], [3]).

Many medusozoans have complex life cycles with the succession of three stages: a short-living larva (*planula*), that metamorphoses into a benthic asexually reproducing polyp stage, giving rise to planktonic adult medusae through lateral budding (Hydrozoa), strobilation (Scyphozoa) or complete metamorphosis (Cubozoa). The presence of benthic polyps may limit the distribution of pelagic jellyfish in coastal areas with available hard substrates for larval settlement and polyp formation [1]. On the other hand, the modularity of a post-larval stage (the polyp) leads to exponential growth of jellyfish populations via polyembryony, i.e. the multiple production of adult (medusa) stages from a single fertilized egg [4]. However, some jellyfish species such as the mauve stinger *Pelagia noctiluca* are holoplanktonic (i.e. do not have a benthic polyp stage), and have a wide distribution in both inshore and offshore waters [5].

*P. noctiluca* has a global distribution [6] and inhabits preferentially warm and temperate waters [7]–[9]. In the Mediterranean Sea, massive blooms of *P. noctiluca* have been reported since the seventeenth century [10], insomuch that the species is almost considered a plague for human activities in coastal waters and has attracted special interest and concern since the late seventies [11]. Aggregations of hundreds thousands, or millions of individuals are not rare for this species whose population abundances show large fluctuations. Indeed, sudden demographical outbreaks lasting one or more years are normally followed by abrupt collapses, apparently with a periodicity of about 12 years [10], [12]. The mechanisms leading to such oscillations are not completely clear. Goy et al. [10] suggested that *P. noctiluca* could take advantage from occasional warm and dry weather conditions during late spring, going toward a demographic explosion in the following summer. This explanation is reasonable, as warm conditions could support the survival of a bigger number of the ephyrae produced by external fertilization of the eggs during spring-summer period [13]. Nevertheless, climatic conditions do not probably represent the only factor in play and further efforts are required to clarify the eco-physiological optima for *P. noctiluca*, identify biological characters and habitat changes apt to boost its outbreaks, distinguish critical thresholds of change, and quantify spatial and temporal levels of probabilities of outbreaks. By identification of reproductive units and measurements of gene flows, population genetics can help to fill some of current gaps of knowledge and advance our understanding of the ecological processes behind jellyfish bloom events.

Previous population genetics and phylogeographic studies focusing on *P. noctiluca* at a large scale in the Mediterranean Sea and Eastern Atlantic Ocean indicated that high level of gene flow allows for a great connectivity across very large areas, maintaining a substantial panmixia [14], [15]. Nevertheless, the peculiar population dynamics of this species in the Mediterranean Sea and in the neighbouring European Atlantic waters suggest the existence of complex underlying mechanisms. Indeed, several factors can play a substantial role in structuring natural populations, resulting sometimes in genetic patterns more complex than expected [16]–[18]. For instance, due to their high census population size many marine animal populations are generally considered not significantly influenced by processes as genetic drift or non-random mating. Moreover, when high population size is accompanied by great dispersal potential, unstructured panmictic populations are expected at a scale lower than the minimum distance dispersal of individuals. Nevertheless, a growing number of studies focusing on species characterised by high dispersal potential, as benthic marine invertebrates with a pelagic larval stage, showed low but significant levels of genetic differentiation among samples taken at distances far below the expected potential dispersal range [19]–[26]. Most of these studies highlighted also a co-occurrence between genetic heterogeneity on a small scale and temporal instability of genetic differentiation among populations, reporting swift fluctuations in time in the form of spatial structure changes across generations, or changes in allelic frequencies at a given sampling point. Such complex spatio-temporal genetic patterns led Johnson and Black [18] to coin the term “chaotic genetic patchiness” (CGP), later paraphrased by David et al. [27] as “fluctuating genetic mosaics”. Different hypotheses have been formulated to explain CGP, but the most widely accepted is that factors linked to the reproductive strategy (e.g. high fecundity and high mortality in early life stages) can lead to a big variance in reproductive success and, consequently, to a reduction of the effective population size [28], [29]. These findings are changing a traditional paradigm, introducing the novel concept that many marine species, even if characterised by high census population sizes, can be exposed to processes usually considered effective only on small populations [30]. Hedgecock [28] compared the reproduction of such species to a sweepstakes lottery, characterised by an unbalanced distribution of the jackpot: “a small number of big winners grab all the prizes, leaving many losers empty-handed”. Projecting this concept to a biological context, it means that stochastic factors can lead to an unbalanced genetic composition of the recruits due to a small number of successful progenitors. Consequence of this kind of processes could be a high level of relatedness between individuals coming from the same area and therefore inbreeding rates higher than expected. Nevertheless, even if many evidences support the existence of CGP processes, few studies have clearly demonstrated a connection between kin aggregation and “fluctuating genetic mosaics” patterns [23], [31]. The aim of the present work is to use a set of newly developed microsatellite markers to study the population genetics of *P. noctiluca* at the small spatial scale of the Southern Tyrrhenian Sea and in a temporal range of 3 years (2010–2012), in order to elucidate the processes behind the genetic composition of each “*bloom unit*”. Indeed, as already mentioned above, this species shows complex population dynamics that may suggest the driving action of unknown factors. Moreover, even if *P. noctiluca* is a holopelagic species, from a genetic point of view it may behave as a typical CGP species: it is characterised by high dispersal ability and the individuals of each *bloom unit* presumably spend together most part of their life, similarly to those belonging to a spatially stable benthic population. This particular habit suggests the possibility that related individuals, born at the same time and in the same area, could later spawn close to each other, producing inbred offspring and a bloom unit structure with high level of relatedness. Considering this hypothesis, the expected genetic patchiness could be further exacerbated by the high mortality rate due to stochastic factors, as unfavourable environmental conditions (i.e. sea storms), or by the high variance in reproduction success due to the external fertilization strategy [32], [33]. The findings of the present work can play a pivotal role for any future investigations to explain the population genetic structure of the species at a broader geographical scale.

## Materials and Methods

### Ethics Statement

*Pelagia noctiluca* is not an endangered species and no special permits were needed for sampling. All the animals were released without serious damages after sampling. Cnidarians are also renowned for their high regeneration potential.

### Study sites and samples collection

**Figure 1:**
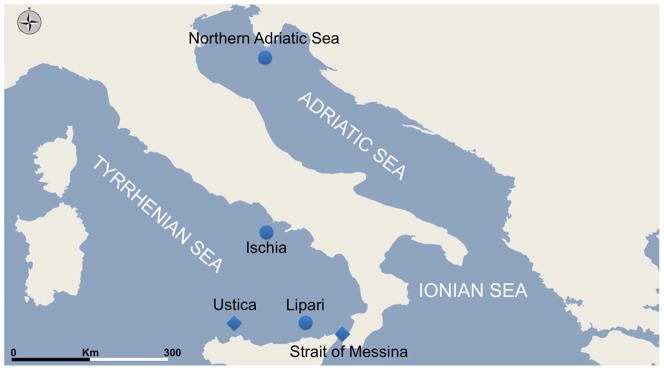
Map of the study locations. Squares indicate locations for which temporal replicates are available; circles indicate location without temporal replicates.

A total of 259 individuals, belonging to the species *Pelagia noctiluca* (Forsskål 1775), were collected from 4 different locations in the Southern Tyrrhenian Sea, and from one site in the Northern Adriatic Sea (see Figure 1 and Table 1, for details on number of individuals analysed for each location, year of collection and coordinates).

**Table 1:** *Pelagia noctiluca* sampling information.

Sampling Site	Sampling Year	Abbreviation	N	Co-ordinates
Northern Adriatic Sea	2006	NAD06	52	44°23′N, 14°44′E
Ustica Island	2010	UST10	43	38°41′N, 13°10′E
Ustica Island	2011	UST11	36	38°41′N, 13°10′E
Ustica Island	2012	UST12	24	38°41′N, 13°10′E
Ischia Island	2010	ISC10	13	40°44′N, 13°56′E
Lipari Island	2011	LIP11	53	38°28′N, 14°57′E
Messina Strait	2011	MES11	14	38°13′N, 15°38′E
Messina Strait	2012	MES12	24	38°13′N, 15°38′E

The table displays sampling sites and collection years, the population sample acronym, the number of individuals and the sampling coordinates.

The collection sites in the Southern Tyrrhenian Sea are rather close to each other (the linear distance between the extreme point of the area is 215 km) and connected by a superficial eastward current [34], [35] likely driving the passive transportation of planktonic organisms across the whole area.

In order to address the species' genetic patterns on both spatial and temporal scale, the sampling was replicated in three consecutive years (2010, 2011, 2012) for Ustica (UST) and in two consecutive years (2011, 2012) for Messina (MES). No temporal replicates were available for the Northern Adriatic Sea (NAD06), Ischia (ISC10), and Lipari (LIP11) locations, respectively sampled only in 2006, 2010 and 2011. The samples were collected by hand-net during bloom events choosing exclusively adults of the same size class, ranging from 7 to 10 cm of bell diameter. For each individual, a small piece of bell margin or oral arm was excised, preserved in 95% ethanol and stored at −20°C until DNA extraction.

### Microsatellite markers development

Total genomic DNA was extracted from the ethanol preserved tissues, following a CTAB-phenol-chloroform based protocol [36], [37].

Microsatellite sequences were isolated by Ecogenics GmbH Switzerland (www.ecogenics.ch) using the high-throughput genomic sequencing approach described by Abdelkrim et al. [38]. Two µg of genomic DNA from 12 *P. noctiluca* individuals belonging to the only two populations already available in 2010, Ustica (UST) and Ischia (ISC), were analysed on a Roche 454 GS-FLX platform (Roche, Switzerland) using 1/8 run and the GS-FLX titanium reagents. The total 53,066 reads had an average length of 277 base pairs. Of these, 843 contained a microsatellite insert with a tetra- or a trinucleotide of at least 6 repeat units or a dinucleotide of at least 10 repeat units. Suitable primer design was possible for 122 reads, 12 of which were tested in agarose by Ecogenics GmbH Switzerland. In order to complement the set of loci, 10 additional reads were selected from the microsatellite library provided by Ecogenics using the software MSATCOMMANDER 1.0.8-beta [39] and primer pairs were designed with Primer3 v.0.4.0 (http://bioinfo.ut.ee/primer3-0.4.0/) [40], [41]. In total, 22 primer pairs were tested on 20 *P. noctiluca* individuals of a single population (UST10), performing PCRs as follows. The PCR volume of 10 µl contained approximately 50 ng of genomic DNA, 1× Mastermix (RBC Taq Polymerase Kit), 0.5 µM of a fluorescently labelled (6-FAM) universal M13 primer (5′CACGACGTTGTAAAACGAC3′) and the species-specific reverse primer, 0.15 µM of species-specific forward primer with a 5′ M13 tail [42], 0.2 mM each dNTP and 1 unit taq. Amplifications were performed separately for each locus in a Eppendorf Mastercycler Gradient using the following thermal cycling profile: 94°C for 10 min, 10 cycles of 94°C for 30 sec, 55°C for 45 sec, 72°C for 45 sec, 25 cycles of 94°C for 30 sec, 53°C for 45 sec, 72°C for 45 sec and 72°C for 10 min. Products were capillary electrophoresed on an ABI 3730XLs by Macrogen (Korea - http://www.macrogen.com) using the internal size standard LIZ 500 (Applied Biosistems). The chromatograms were analysed using GeneMarker v. 2.2.0 (SoftGenetics) and a final panel of nine loci was obtained after excluding loci with low signal, unclear peaks, excessive stuttering and the non-polymorphic ones.

### Markers genotyping

Two multiplex panels were created with the help of the software Multiplex Manager 1.0 [43] setting 5 as maximum number of loci per reaction, 6 as primers complementarity threshold and 40 bp as minimum distance between loci of the same dye colour. Forward primers were labelled with different fluorescent dyes (6-FAM, VIC, NED – Applied Biosystems) and PCRs were carried out by multiplexing between 4 and 5 loci (multiplex content in Table 2) in 10 µl total volume containing: 5 µl of Qiagen Multiplex PCR Master Mix, 2 µl of Q solution (Qiagen), 0.2 µM of each primer, and 50 ng of genomic DNA. PCR profile followed manufacturer's instructions and annealing temperature was set at 57°C for each multiplex reaction. The products of each multiplex PCR were electrophoretically separated on an ABI 3730XLs by Macrogen (Korea- http://www.macrogen.com) using the internal size standard LIZ 500 (ABI). Re-extraction and repeated multiplex amplifications were performed on individuals with failed PCRs. In addition, to assess loci amplification and scoring repeatability, 10% of total sampled individuals were randomly re-amplified and alleles sized [44]. Allele sizes were assigned using GeneMarker v. 2.2.0 (SoftGenetics) and allele scoring was repeated independently by two authors/operators and then compared. Binning was automated with the software Flexibin ver. 2 [45]. All input files for further analysis were produced with the software Create [46].

**Table 2:** General information about microsatellite markers in *Pelagia noctiluca*.

Locus	Repeat Motif	Genbank Acc. No.	Primers sequence (5′-3′)^a^	Size Range (bp.)	Primer label^b^	Multiplex Panel^c^	T Annealing (°C)
**Pelnoc_40622**	(TCA)8	KF896613	CGTGGTACTTCATCATGTGGC TTCACAACAGCCATTCACGC	117–135	6-FAM	1	57
**Pelnoc_39456**	(CT)10	KF896614	TTGCCTGACATAAGCTCTACG CAACTACCTCGTCGACCCTC	179–229	6-FAM	1	57
**Pelnoc_46263**	(CA)12	KF896615	CGCTGGTCCGATCATTTATCC TGGGCCTACGAATTAAGGAGG	261–319	6-FAM	1	57
**Pelnoc_44003**	(AAC)7	KF896616	ATGCCATGAATTCGGGTTCC TAGGACCCGTAGCGTTTTCC	158–191	VIC	1	57
**Pelnoc_44210**	(TGT)9	KF896617	GGGGGTACTGACCCAGTTG TTTACGTATGGCCAGCATCA	139–169	6-FAM	2	57
**Pelnoc_40428**	(TTG)8	KF896618	GCCGTGTCACCTTCATTCTG CCCCTTTGTGAATCTGAACC	202–256	6-FAM	2	57
**Pelnoc_40199**	(CT)8	KF896619	GCTGCAGTTTGGTTGTGCTA TAGGAGCGCATTGCTGTAAA	301–385	6-FAM	2	57
**Pelnoc_16756**	(TGT)7	KF896620	TACGGCTTGTCTCCCGTATG GGATTCCGATGCCGTTTAGC	206–239	VIC	2	57
**Pelnoc_7445**	(TTG)8	KF896621	TTACAACCTGCACACAAGCG TTACTACCTTACGTGCCCCC	126–150	NED	2	57

a For each locus, the upper sequence refers to the forward primer, the lower sequence refers to the reverse primer.

b Label at the 5′– end of the forward primers.

c Loci showing the same number were amplified together in the same multiplex PCR.

### Markers characteristics and within population genetic variation

Levels of polymorphism were calculated for each locus in each population. Number of alleles (N_A_) was obtained using the software FSTAT v. 2.9.3.2 [47], while observed and expected heterozygosity (H_O_; H_E_) were calculated by GENETIX v. 4.05.2 [48].

Microsatellite loci are informative when they are independent one another and do not produce redundant information, therefore linkage disequilibrium was tested for each pair of loci within each population using Fisher's exact tests by Genepop web version [49], [50] (http://genepop.curtin.edu.au/). The corrected significance threshold for multiple tests was set using the Bonferroni procedure [51], [52].

As mentioned, the dataset was carefully checked against inconsistencies due to human mistake, genotyping errors and amplification failure. However, in order to evaluate and minimise the presence of artefacts, the final dataset was analysed with Microchecker v. 2.2.3 [53] and null allele frequencies estimated with the correction algorithm of van Oosterhout et al. [53]. Null alleles are a common feature of the microsatellites markers and are often accountable for an increased observed homozygosity. The software adjusts the number of homozygote genotypes in each size class to reflect the estimated frequency of null alleles and the “real” number of homozygotes. Therefore, a new dataset of each locus was obtained by considering the adjusted genotypes and, whenever possible, it was used simultaneously with the original one. The effect of the possible presence of null alleles was evaluated comparing the results obtained using the two datasets.

In order to verify the effect of the correction for null alleles on HWE, two parallel analyses (with and without correction for null alleles) were performed with Arlequin ver. 3.5.1.3 [54], and respective p-values of HWE were calculated implementing an exact test with 1,000 steps in Markov chain and 10,000 dememorization steps. Moreover, the same strategy was used to calculate the Weir and Cockerham [55] inbreeding indices (F_IS_) using FSTAT [47]. Significance levels for the global F_IS_ values were calculated by performing 1000 randomizations of genotypes among samples.

High F_IS_ values and deviation from HWE expectations may be interpreted as an evidence of null alleles presence. Nevertheless, the Hardy-Weinberg disequilibrium (HWD) can be also due to phenomena as Wahlund effect [56] or non-random mating, especially when the HWD is due to heterozygotes deficiency and is associated with highly positive inbreeding values. The comparative approach with and without null alleles correction employed in this study aims to exclude null alleles pervasiveness.

### Relatedness patterns

The degree of relatedness between individuals of the same “bloom unit” was firstly investigated using the software ML-Relate [57]. Remarkably, this software allows testing for the presence of null alleles, as indicated by a deficiency of heterozygotes relative to Hardy–Weinberg expectations [58], and eventually accommodating them in the subsequent analyses of relatedness. ML-Relate was therefore used employing a maximum likelihood approach to calculate pairwise Wright's [59] coefficients of relatedness (r) and the specific patterns of relationship (R) between individuals, classified as unrelated (UR), half-sib (HS) or full-sib (FS) and ranked depending on their likelihood values [ML(R)]. In order to verify if the presumed presence of null alleles increased the estimated relatedness between individuals within and among population samples pairs (inter- and intra-population r), two parallel analyses were performed with and without correction for null alleles. Relatedness values were obtained for all individual pairs (with and without accounting for null alleles) for the whole dataset of 259 specimens and used to calculate an average relatedness coefficient for each population pair. A two tailed t-test was then applied to verify if r-values obtained without correcting for null alleles were significantly different than those calculated taking null alleles into account. In addition, to verify if, by chance, intra-population r-values could have been equal or higher than observed, a Monte Carlo Markov Chain (MCMC) randomization of r coefficients was also applied calculating new inter- and intra-population r, after 1000 permutation of individuals across populations using PopTools [60]. Finally, a ranking of the most likely relationship was calculated for each individual pair within each population sample, and the frequency of pairs resulting to be FS and HS was estimated. Frequencies were calculated for both corrected and uncorrected datasets and compared by means of a two tailed t-test.

Since every method of parental analysis and sibship reconstruction includes a certain level of uncertainty ([61] and references therein), additional analyses were performed with the software Colony v. 2.0.4.1 [62]. Indeed, comparing the results of independent analyses performed with different programs is a strategy that can highlight possible failings in case of discordant results or ensure more robustness to agreeing outcomes. The statistical significance of differences between the two software's outcomes was assessed using a two-tailed t-test.

The maximum full-likelihood method implemented in Colony v. 2.0.4.1, was used to partition dyads as HS or FS and to infer family structures within the “bloom units”. Since Colony associates a probability value to each result, only those with a probability higher or equal to 95% were taken into account. Separate runs were performed for each sample, using the inbreeding model, as suggested for dioecious species when the inbreeding level is high (cf. Colony user guide), and setting medium length of the run and high likelihood precision. Due to the lack of prior information about sibship size, the value 0 was set up for known male and female genotypes as well as for known paternal and maternal sibship. Since the program Colony can accommodate null alleles and other stochastic genotyping errors in the analysis, once again two runs were performed for each sampling site, taking or not the presence of null alleles into account. In the runs “without nulls”, the genotyping error was set to the default level (0.005), whereas the runs “with nulls” were performed allowing locus specific genotyping error levels, obtained adding the null alleles frequencies calculated by Microchecker v. 2.2.3 for each locus in each population to the default genotyping error rate. HS and FS dyads frequencies were calculated for both corrected and uncorrected datasets and then compared by means of a two tailed t-test.

Family structures were inferred reconstructing extended sibship networks, namely clusters of individuals connected by a chain of HS or FS intermediate individuals. In practice, if the individual A shares a parent with the individual B, and the individual B shares a parent with the individual C, in that case A and C are linked through B and are members of the same extended sibship network, even if they do not have a common parent. Since the extended sibship networks can also include FS, they were represented as FS families (groups of individuals sharing both the parents) nested into sibship networks. For each sibship network the number of FS families (and the respective number of family members) was reported, when any.

### Population structure

To examine patterns of genetic variation among the studied population samples, pairwise F_ST_ values and corresponding p-values were calculated with Arlequin ver. 3.5.1.3. Prior to this analysis, in order to estimate the extent of bias possibly introduced by the presence of null alleles, pairwise F_ST_ were also calculated using the software FreeNA [63] with and without null alleles correction. This software, indeed, estimates null allele frequencies for each locus and population, following the Expectation Maximization (EM) algorithm of Dempster et al. [64] and then implements the so-called ENA correction to provide accurate estimation of F_ST_ [65] in presence of null alleles. 95% confidence intervals for the F_ST_ values were obtained using 50,000 bootstrap iterations. F_ST_ estimates obtained with and without applying the ENA algorithm were compared by means of a two tailed t-test.

## Results

### Markers characteristics and within population genetic variation

Of 22 initially selected loci, 13 were not polymorphic or showed very low signal, not clear peaks or excessive stuttering and were discarded, while 9 markers were suitable for the present study and resulted to be polymorphic in all population samples (Table 2). The total number of alleles ranged from 5 to 18 (Mean = 8, Standard Deviation SD = 3.7) while observed heterozygosity ranged from 0.14 to 0.85 (Mean = 0.48, Standard Deviation SD = 0.16) (Table 3).

**Table 3:** Summary of genetic variability for all *Pelagia noctiluca* samples.

Locus	Population															
	ISC10				UST10				LIP11				MES11			
	NA	Ho	He	HWE	NA	Ho	He	HWE	NA	Ho	He	HWE	NA	Ho	He	HWE
**Pelnoc_40622**	2	0.153	0.270	0.234	4	0.186	0.175	1.000	4	0.264	0.269	0.730	3	0.143	0.140	1.000
**Pelnoc_39456**	7	0.700	0.847	0.329	7	0.317	0.671	**P<0.0001**	14	0.489	0.833	**P<0.0001**	6	0.429	0.724	**0.020**
**Pelnoc_46263**	11	0.615	0.886	**0.027**	16	0.534	0.894	**P<0.0001**	15	0.585	0.877	**P<0.0001**	9	0.714	0.843	0.149
**Pelnoc_44003**	4	0.333	0.634	**0.028**	4	0.405	0.524	**P<0.0001**	6	0.283	0.537	**P<0.0001**	3	0.429	0.442	1.000
**Pelnoc_44210**	5	0.692	0.796	0.169	7	0.400	0.814	**P<0.0001**	9	0.547	0.800	**0.001**	6	0.714	0.767	0.558
**Pelnoc_40428**	4	0.307	0.396	0.526	6	0.286	0.427	**P<0.0001**	8	0.377	0.515	0.034	6	0.429	0.487	0.622
**Pelnoc_40199**	9	0.615	0.880	0.089	15	0.524	0.852	**P<0.0001**	18	0.472	0.880	**P<0.0001**	10	0.538	0.883	**P<0.0001**
**Pelnoc_16756**	5	0.692	0.646	0.647	7	0.511	0.617	0.187	7	0.528	0.589	0.030	6	0.571	0.653	0.165
**Pelnoc_07445**	6	0.818	0.749	0.182	8	0.767	0.675	0.951	8	0.529	0.751	**P<0.0001**	6	0.857	0.807	0.926

Locus	Population															
	UST11				MES12				UST12				NAD06			
	NA	Ho	He	HWE	NA	Ho	He	HWE	NA	Ho	He	HWE	NA	Ho	He	HWE
**Pelnoc_40622**	3	0.286	0.252	1.000	3	0.250	0.582	**P<0.0001**	5	0.125	0.607	**P<0.0001**	4	0.192	0.298	**0.003**
**Pelnoc_39456**	13	0.444	0.840	**P<0.0001**	12	0.522	0.796	**P<0.0001**	12	0.667	0.871	**P<0.0001**	9	0.346	0.735	**P<0.0001**
**Pelnoc_46263**	13	0.750	0.885	0.150	10	0.625	0.855	**0.014**	13	0.667	0.883	**0.004**	15	0.481	0.876	**P<0.0001**
**Pelnoc_44003**	6	0.333	0.593	1.000	3	0.292	0.377	**0.025**	2	0.458	0.439	1.000	6	0.461	0.560	0.064
**Pelnoc_44210**	9	0.471	0.845	0.558	7	0.391	0.813	**P<0.0001**	7	0.500	0.777	**0.013**	8	0.510	0.812	**P<0.0001**
**Pelnoc_40428**	8	0.361	0.411	0.622	7	0.292	0.339	0.236	6	0.375	0.398	0.350	9	0.423	0.505	**0.015**
**Pelnoc_40199**	15	0.657	0.872	**P<0.0001**	10	0.583	0.887	**P<0.0001**	14	0.625	0.914	**0.004**	14	0.380	0.888	**P<0.0001**
**Pelnoc_16756**	7	0.611	0.605	0.165	6	0.500	0.525	0.794	6	0.625	0.613	0.831	6	0.519	0.560	0.542
**Pelnoc_07445**	8	0.543	0.643	0.926	7	0.500	0.637	0.087	7	0.500	0.608	**0.010**	10	0.481	0.671	**P<0.0001**

Eight sampling sites at nine microsatellite loci are described, including total number of alleles (NA), observed (Ho), expected heterozygosity (He) and probabilities of deviation from Hardy–Weinberg equilibrium (HWE).

a Values in bold indicate significant HWE deviations (α = 0.05).

Microchecker did not identify scoring errors associated with stuttering, but suggested the presence of null alleles by analysing strong deviations from HWE (Table 3). Indeed, all loci showed heterozygote deficit in at least 3 out of 8 populations, except for the locus Pelnoc_16756 having a moderate homozygote excess only in one population (LIP11, P = 0.030). Loci potentially affected by null alleles over all populations are showed in Table S1.

Two out of 36 pair-wise locus comparisons revealed significant linkage disequilibrium, after standard Bonferroni adjustment [51], [52], between loci Pelnoc_46263 vs. Pelnoc_40199 and Pelnoc_46263 vs. Pelnoc_7445 at UST10 only. This result might be due to lack of recombination, non-random mating in inbred populations or admixture of genetically distinct populations (i.e. Wahlund effect [56]), all factors able to cause loci to appear statistically linked [66].

All loci but one (Pelnoc_16756; P-value = 0.290) did not globally result in HWE. HWD was due to heterozygote deficiency for all cases, suggesting two alternative explanations: the presence of technical artefacts, such as non-amplifying alleles (null alleles), or the influence of biological factors [67]. However, given the strong deviation from HWE observed in almost the whole set of loci, null alleles specifically affecting each single locus seem unlikely to be the only factor responsible for the observed pattern of disequilibrium. In addition, if null alleles only were involved, the HWD should disappear after correction using Microchecker (see methods). Indeed, although for some loci in some populations the extent of HWD for the corrected dataset was lower (HWE p-values are shown in Table S2), globally the HWE was not recovered. In fact, only for 14 out of 41 test showing HWD (locus by locus estimation) the equilibrium was re-established after applying the correction for null alleles.

Inbreeding coefficient estimation confirmed the significant excess of homozygotes, with F_IS_ values generally higher than zero (Table 4). Similarly to what observed for the HWE comparisons, inbreeding estimates calculated using the dataset corrected for null alleles were still positive (average 0.087), although lower than those calculated with the original, uncorrected, dataset (average 0.301).

**Table 4:** F_IS_ values calculated with and without null alleles correction.

FIS Estimates				
Locus	Uncorrected	95% C.I.	Corrected	95% C.I.
**Pelnoc_40622**	0.317		−0.008	
**Pelnoc_39456**	0.428		0.091	
**Pelnoc_46263**	0.318		0.112	
**Pelnoc_44003**	0.310		0.122	
**Pelnoc_44210**	0.380		0.040	
**Pelnoc_40428**	0.201		0.100	
**Pelnoc_40199**	0.414		0.149	
**Pelnoc_16756**	0.069		n.e.	
**Pelnoc_07445**	0.150		0.051	
**TOT**	**0.301**	0.214–0.371	**0.087**	0.058–0.113

Locus by locus and over all loci F_IS_ values were calculated for the original and the corrected dataset after Microchecker v. 2.2.3 analysis.

n.e.: Not evaluated due to HWE and no changes in the corrected dataset.

The observed heterozygosity lower than expected at almost all loci, the presence of sporadic linkage disequilibrium in one single population, and the persistence of HWD and positive F_IS_ values after correction for null alleles represent altogether strong complementary indications that true biological factors, rather than technical artefacts only, are responsible for the observed pattern of disequilibrium.

### Relatedness patterns

Average relatedness between individuals within and among Southern Tyrrhenian population samples was investigated using ML-relate with and without correction for null alleles. The two-tailed t-test applied to compare r-values obtained with and without correction resulted to be significant (P<0.0001) due to higher r-values obtained taking into account the presence of null alleles (Relatedness Monte Carlo Simulations are shown in Tables S3 and S4). However, a higher than expected relatedness among individuals of several samples was detected that was robust against null allele correction (Table 5). Indeed, Monte Carlo simulation tests indicate that 4 out of 8 intra-population pairwise comparisons resulted to have a significantly higher r-value than expected by chance when not taking into account null alleles presence (Table 5). Correcting for null alleles resulted in a small difference and 3 out of 8 significant comparisons remained significant after the correction. Considering conservatively the results obtained with null alleles correction, our approach suggested that at least UST10, MES11 and MES12 have higher within-population relatedness degree than expected by chance.

**Table 5:** Relatedness index p-values after Monte Carlo simulation testing.

A							
POP	UST10	UST11	UST12	ISC10	LIP11	MES11	MES12
**UST10**	**P<0.0001**						
**UST11**	**0.035**	0.936					
**UST12**	0.989	0.720	0.204				
**ISC10**	0.232	0.399	0.555	0.541			
**LIP11**	0.189	0.752	0.992	**0.019**	0.748		
**MES11**	0.541	0.972	0.985	0.216	0.978	**0.046**	
**MES12**	0.433	0.818	**P<0.0001**	0.761	0.995	0.889	**P<0.0001**

B							
POP	UST10	UST11	UST12	ISC10	LIP11	MES11	MES12
**UST10**	**P<0.0001**						
**UST11**	**0.009**	0.975					
**UST12**	0.997	0.800	**0.049**				
**ISC10**	0.254	0.241	0.490	0.527			
**LIP11**	**0.036**	0.447	0.998	**0.016**	0.556		
**MES11**	**0.295**	0.936	0.999	0.460	0.989	**0.049**	
**MES12**	**0.634**	0.996	**P<0.0001**	0.918	**0.999**	0.953	**0.002**

Probability that relatedness index (r) could be higher than observed within the population samples pairs. A: Taking into account the presence of null alleles; B: Not considering null alleles. Intra-population comparisons are in diagonals. Significant values are in bold. All relatedness values are reported in Tables S3 and S4.

When using ML-Relate to estimate the parentage relationships among individuals (Table 6A), all samples, except ISC10, MES11 and UST12, were shown to contain some related individuals. The highest frequencies of HS and FS pairs were present in LIP11 (HS = 0.03544; FS = 0.00452) and in NAD06 (HS = 0.03619; FS = 0.00150). Moreover, the amount of HS and FS pairs calculated with and without accounting for null alleles was not significantly different (p-values>0.30), clearly indicating that this result cannot be explained by non-amplifying alleles.

**Table 6:** Frequencies of Half Sib and Full Sib dyads estimated by A) ML-Relate and B) Colony.

A				
Populations	Frequencies			
	HS _NNA	HS_NA	FS_NNA	FS_NA
**NAD06**	0.03619	0.02488	0.00150	0,00301
**UST10**	0.01357	0.01432	0.00226	0,00226
**UST11**	0.01131	0.00980	0.00000	0,00075
**UST12**	0.00000	0.00075	0.00075	0,00075
**ISC10**	0.00000	0.00000	0.00000	0.00000
**LIP11**	0.03544	0.03544	0.00452	0,0015
**MES11**	0.00000	0.00000	0.00000	0.00000
**MES12**	0.00377	0.00301	0.00075	0,00075
p-values	0.3250		0.8436	

B				
Populations	Frequencies			
	HS_NNA	HS_NA	FS_NNA	FS_NA
**NAD06**	0.04444	0.06570	0.00580	0.00097
**UST10**	0.04858	0.07152	0.00539	0,00404
**UST11**	0.03968	0.06507	0.00000	0.00000
**UST12**	0.02766	0.06719	0.00000	0.00000
**ISC10**	0.00000	0.00000	0.00000	0.00000
**LIP11**	0.03338	0.04789	0.00290	0,00072
**MES11**	0.00000	0.01282	0.00000	0.00000
**MES12**	0.04710	0.47101	0.00724	0,00724
**p-values**	0.2096		0.1839	

HS: half-sib dyads; FS: full-sib dyads; NNA: no null alleles accounted for; NA: null alleles accounted for.

In each table, the last row reports the p-values of the t test comparing each category of estimated frequencies (e.g. FS “with nulls” vs. FS “without nulls”).

The results of the analyses of parentage relationships performed by Colony show agreement with those obtained using ML-Relate by disclosing the existence of related individuals in many of the investigated populations. Separate approaches are used by the two software: Colony jointly considers the likelihood of larger patterns of relationship, whereas ML-Relate independently determines the relationship of each pair of progeny [68]. Therefore, some differences were also found. In particular, HS dyads were found by Colony in all samples except ISC10 and MES11, while FS dyads were found in UST10, LIP11 MES12 and NAD06 (Table 6B). Unlike the ML-Relate results, the highest HS dyads frequencies are present in UST10 (0.04858), while the highest FS pairs frequencies are shown by MES12 (0.00724). Also in this case, the comparison between relationships calculated with and without accounting for null alleles was not significant (P>0.18), whereas the two tailed t-test between the ML-Relate and Colony outcomes showed a significant difference only between HS frequencies (P = 0.028) due to a higher average of HS frequencies found by Colony (Mean = 0.031, Standard Deviation SD = 0.019) respect to ML-Relate (Mean = 0.013, SD = 0.015).

**Figure 2:**
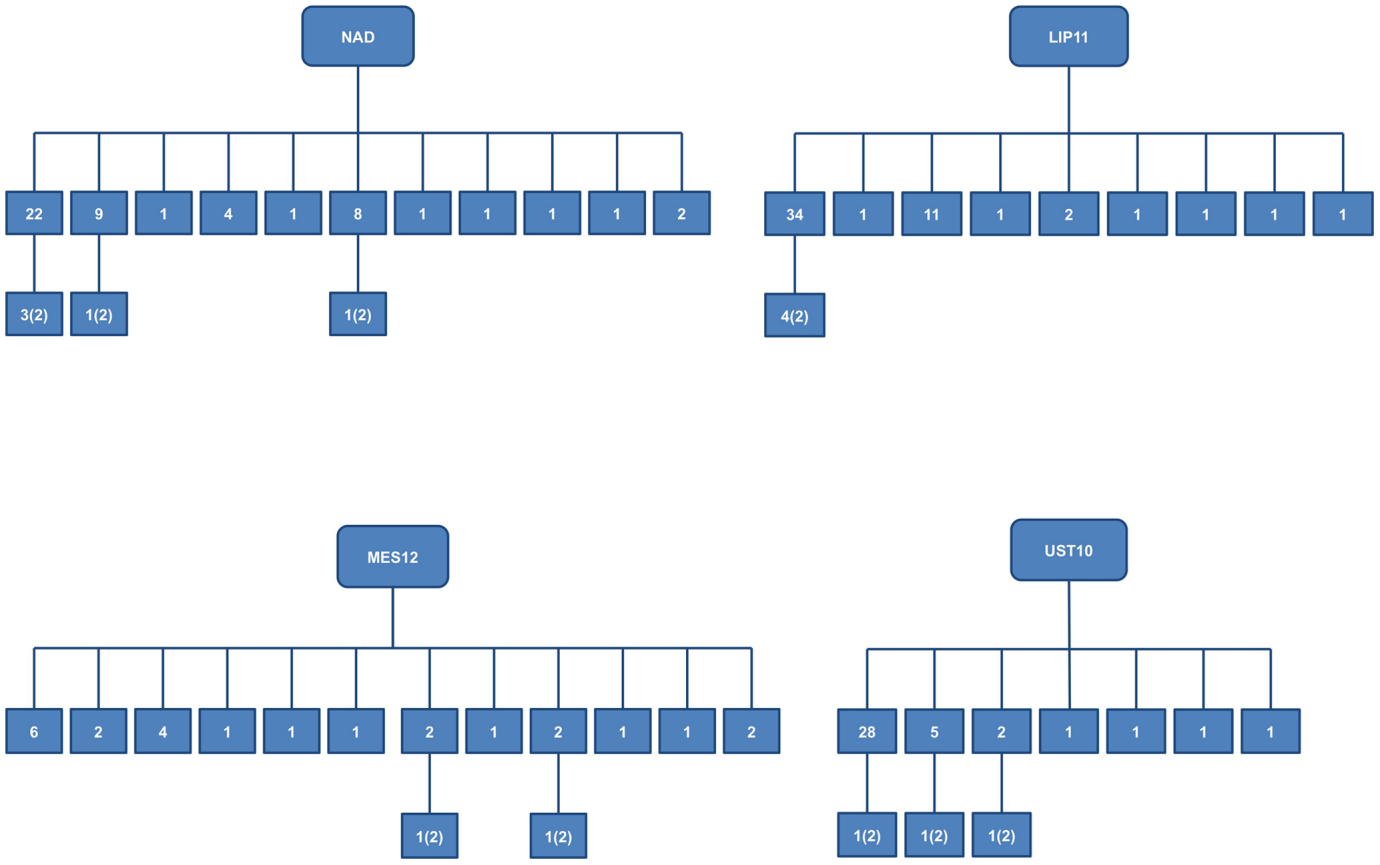
Family structure in four *Pelagia noctiluca* Mediterranean populations. The first line of each dendrogram shows the extended sibship networks produced by Colony v. 2.0.4.1. For each population, in this line the squares indicate the number of individuals linked by kinship (directly or indirectly). For example the number “34”indicates that 34 individuals are linked together by kinship. Separate squares refer to individuals with no connections. The second line shows the Full Sib (FS) families nested in the sibship network. Namely, since each network includes all the individuals linked by a certain level of kinship (individuals sharing one or two parents), when any, in the second line the FS individuals (sharing both parents) were reported, specifying the number of families and the respective number of family members (in brackets). For example, the numbers “4(2)” indicate the presence in the upper sibship network of 4 FS families composed by 2 individuals each.

As regards family reconstruction with Colony, results were consistent among replicates. Since the results of the analyses with and without accounting for null alleles were not significantly different, only the results of the runs “without nulls” are shown. The more structured families (containing both HS and FS individuals) were found in UST10, LIP11, MES12 and NAD06, with respectively 3 FS families nested within 7 sibship networks, 4 FS families nested within 9 sibship networks, 2 FS families nested within 12 sibship networks and 5 FS families nested within 11 sibship networks. All FS families were composed by two individuals. A graphic representation of the family structures found in these 4 populations is shown in Figure 2. Relating to the other investigated populations, Colony identified in UST11 a single sibship network composed by 28 individuals, while other 7 individuals resulted unrelated two sibship networks, respectively of 10 and 5 individuals, were identified in UST12, paralleled by 8 unrelated individuals. None of the latter two populations showed FS families, whereas the populations ISC10 and MES11 did not show any kind of family structure.

### Population structure

Population pairwise F_ST_ values were initially calculated with FreeNA to verify if accounting for null alleles could bias this genetic distance estimation (Table S5). F_ST_ values calculated with and without applying the ENA correction resulted to be significantly different due to an increase of F_ST_ distances when accounting for null alleles. The occurrence of null alleles produces an overestimation of the F_ST_ values in case of significant population differentiation, which is dampened by corrections for nulls [63]; therefore, only uncorrected F_ST_ values were considered, believing they still represent the best estimations for a conservative analysis of population differentiation. Accordingly, the global and the pairwise F_ST_ values calculated with Arlequin ver. 3.5.1.3 from the original dataset were taken into account for further consideration. The overall F_ST_ value was small (0.01714, 95% CI 0.00501–0.03949) but highly significant (P<0.0001). Inspection of pairwise F_ST_ values (Table 7) indicated that the overall differentiation found is mainly attributable to the sample collected at Messina in 2012, which was significantly different in 5 out of the 7 test performed. Surprisingly, this differentiation greatly exceeds that of the sample from Northern Adriatic Sea (NAD06).Despite of more than a thousand kilometres of geographical separation from the Tyrrhenian samples and a different year of collection (2006 against 2010–2012), they resulted to be significantly differentiated only in 2 out of 7 test, whereas all the other comparisons were not significant (P≥0.06934). Interestingly, the distinctiveness of the MES12 sample does not seem to reflect stable geographic differentiation, given that MES12 was different from the sample MES11, collected one year before at the same location (MES11-MES12 F_ST_ = 0.05338, P<0.0001), and homogeneous with the sample UST11, collected in 2011 at ≈230 Km of distance (UST11-MES12 F_ST_ = 0.00184, P = 0.90137). Moreover, temporal variation was identified also at the Ustica site (UST10-UST12 F_ST_ = 0.04007, P<0.0001).

**Table 7:** Pairwise F_ST_ and respective p-values.

POP	NAD06	UST10	UST11	UST12	ISC10	LIP11	MES11	MES12
**NAD06**		0.0762	0.1211	**P<0.001**	0.5771	0.1445	0.0693	**P<0.001**
**UST10**	0.0090		0.2960	**P<0.001**	0.1963	0.1943	0.0205	**P<0.001**
**UST11**	0.0079	0.0054		0.0195	0.9893	0.9355	0.0605	**P<0.001**
**UST12**	**0.0324**	**0.0401**	0.0167		0.1719	**0.0010**	0.0019	0.9014
**ISC10**	0.0045	0.0129	0.0000	0.0157		0.9775	0.2471	0.0049
**LIP11**	0.0064	0.0059	0.0000	**0.0235**	0.0000		0.1318	**P<0.001**
**MES11**	0.0192	0.0246	0.0171	0.0488	0.0128	0.0149		**P<0.001**
**MES12**	**0.0376**	**0.0480**	**0.0299**	0.0000	0.0410	**0.0381**	**0.0534**	

F_ST_ values for the original uncorrected dataset calculated with Arlequin ver. 3.5.1.3 (Excoffier and Lischer 2010). F_ST_ values are reported below the diagonal, while p-values are shown above the diagonal. In bold, significant values, after Bonferroni correction (α = 0.00179).

## Discussion

*Pelagia noctiluca* blooming populations in the Southern Tyrrhenian Sea exhibited significant deviation from HWE due to large excess of homozygotes for 8 out of 9 microsatellite loci, leading to high inbreeding coefficients (F_IS_). Moreover, higher relatedness than expected by chance, between individuals within and among population samples was detected and supported by the presence of half-sib (HS) individuals in at least 5 out of 8 samples, 4 of which (at least) contained full-sib (FS) individuals also. The genetic differentiation among samples was globally small, but it highlighted a spatial and temporal genetic patchiness probably reflecting the influence of reproductive processes, as also suggested by the outcomes of the relatedness analyses.

In most studies of population genetics, large deviations from HWE are expected to be strongly linked to null alleles, and for this reason several markers are frequently discarded. Although the occurrence of some null alleles in microsatellite markers cannot be completely ruled out, however HWD can also have biological explanations, especially if the deviations are chronic at multiple loci [69], [70]. In the present study, all loci but one strongly deviated from HWE expectations. Therefore, even considering the presence of null alleles, it is unlikely the only factor accountable for the HWD and the positive F_IS_ values, as supported by the missing recovery of HWE (27 out of 41 locus by locus evaluations remained in HWD after null alleles correction, Table S2) and by the still positive F_IS_ values after the null alleles correction (Table 4). Moreover, if null alleles number of blanks was observed in the loci Pelnoc_44210 and Pelnoc_39456 (respectively 9 and 8 blanks over 259 individuals) while all the other loci showed a number of blanks ranging from 0 (Pelnoc_46263) to 5 (Pelnoc_07445) with an average number of 2 blanks over 259 individuals. According to the estimated frequencies of null alleles, for example the locus Pelnoc_46263 should show at least five blanks instead of the zero observed, as well as the locus Pelnoc_40199 showed only three instead of the expected nine blanks; on average 4 null homozygotes should be observed for each locus (data not shown). Basing on this line of reasoning and considering also the sporadic linkage disequilibrium observed in one single population (UST10) as a possible symptom of non-random mating in inbred populations [67], null alleles can be reasonably excluded as the only cause of the strong HWE. Conversely, available evidence conveys links between the observed HWE and biological factors, as supported by the high intra-population relatedness degree (Table 5) and by the occurrence of related individuals in several sampled populations (Table 6), that were found both considering or not the presence of null alleles.

*P. noctiluca* is a species with external fertilization that reproduces during spring and summer [71], [72]. Broadcast spawning in the water column would theoretically ensure homogeneous distribution of gametes and random fertilization, but reproductive and behavioural features may drive patterns of kin aggregation. This seems to be the case for *P. noctiluca*. First, each mature female jellyfish spawns oocytes in a sticky mucus ribbon, holding eggs together for several minutes before its dissolution [72]. This peculiarity may favour fertilization of the whole set of oocytes by sperms released by a single or a few male mates, producing a large amount of full sibs. Second, fusion of gametes produced by related individuals and the resultant formation of inbred offspring may be favoured by aggregative swimming behaviour of jellyfish. Indeed, *P. noctiluca en-masse* proliferations can be characterised by high densities active aggregations routinely exceeding 100 individuals per cubic meter, driven as a whole by surface winds and marine currents [73], [9], [11]. Related individuals born from the same parental group at the same time have a reasonable probability to remain together in the native bloom unit also during subsequent spawning events [74], increasing the probability to produce inbred offspring.Indeed, *P. noctiluca* jellyfish display an active swimming behaviour during daily vertical migration, in response to a corresponding circadian migration pattern of their zooplankton prey [75]–[77]. Canepa et al. [78] also suggested the occurrence of a seasonal migratory circuit along vertical corridors in the proximity of marine canyons, acting as circulation pumps of water bodies favouring local jellyfish aggregations. Physical oceanographic models suggest that water circulation is characterised by both downwelling and upwelling events, influencing nutrient exchange, biological productivity and eventually the composition of shallow and deep-sea biota [79]–[81]. Enhanced upwelling near canyon areas provides increased nutrient exchange that boosts phytoplankton and, hence, zooplankton abundance. Available evidence suggests indeed that submarine canyons have important effects on coastal marine ecosystems, including food webs [79], by acting as additional drivers of environmental and biological discontinuities of the coastal habitats. *Pelagia noctiluca* aggregations are known as strongly influenced by marine currents and favoured by physical discontinuities as fronts and pycnoclines [73], [78], [82]. The up- and downwelling currents driven by marine canyons may therefore represent a driving force for dense aggregations, for both physical (jellyfish pooled together by water movements) and trophic (local increasing of preys availability) reasons [78].

Interestingly, an atypical swimming behaviour of *P. noctiluca* jellyfish has been also reported throughout spring and summer months [78], with frequent formation of jellyfish couples which may presumably boost fertilisation rates as well as full-sibs generation. Living in swarms can provide strong advantages to jellyfish not only facilitating conspecific gametes to fuse, but also allowing highly synchronised reproduction among conspecifics to enhance fertilisation success. Furthermore, although many marine reproductive cycles appear to be on lunar, circadian or circatidal rhythms, waterborne chemical cues are crucial for fine-tuning spawning synchrony [83], [84].

Comparably, at least in some fish species, individuals can remain together from birth to settlement, despite relatively long planktonic durations. Sensorial and behavioural mechanisms enable fish larvae to remain in close proximity of each other throughout their planktonic dispersal and achieve genetically homogeneous recruitment [85]. Finally, individual dispersal in several marine taxa characterised by a pelagic larval phase, including sponges, echinoderms, molluscs, crustaceans, corals and fishes, may be influenced by oceanographic conditions [86]–[93]. In *P. noctiluca* the maintenance of kin-related jellyfish aggregation along marine currents may be similarly influenced by small-scale hydrodynamic and oceanographic patterns conducive to limited individual mixing despite high dispersal potential. Hydrogeographic features such as eddies, gyres or upwelling fronts could restrict dispersal of groups of medusae in confined areas [82], preventing an extensive mixing with individuals belonging to other aggregations and allowing the fusion between gametes produced by related individuals. Consistently with this hypothesis, Lee et al. [94] recently suggested oceanographic barriers to dispersal causing genetic differentiation among some geographically near (≈200 Km) populations of jellyfish *Rhizostoma luteum* in the Irish Sea. Even if the bentho-pelagic life cycle of this species may favour the retention of medusae in coastal areas, a substantial genetic homogeneity should be expected at low spatial scale, unless to consider specific water circulation patterns as hindrance to gene flow.

Additional evidences of biological factors influencing the *P. noctiluca* genetic structure come from population differentiation analyses. According to classical genetic theory, a marine species with high pelagic dispersal potential is expected to have no clear genetic structure at least below the minimum dispersal distance of individuals [95]–[98] and previous phylogeographic studies on *P. noctiluca* confirmed this expectation. Indeed, Miller et al. [15], in a phylogeographic study performed using mitochondrial cytochrome oxidase subunit I (COI) and two nuclear internal transcribed spacers (ITS1 and ITS2) genes, found high and statistically significant genetic differences (at two out of three markers: COI: Φ_ST_ = 0.72, P<0.001; ITS2: Φ_ST_ = 0.023, P<0.001) only between Southern and Northern Atlantic samples, geographically very far from each other (≈10.000 km), whereas no genetic difference was found at a smaller geographic scale. The authors interpreted this result as a suggestion of historical rather than contemporary gene flow. Conversely, Stopar et al. [14] provided support for present-day panmixia among *P. noctiluca* populations from the Mediterranean Sea and the North-western Atlantic Ocean. These authors could not detect significant genetic differentiation relative to both COI and IST1/ITS2 markers, apart from differences between samples from Northern and Southern Adriatic Sea (COI: Φ_ST_ = 0.095, P<0.01). Such a short-scale pattern of genetic differentiation is not coherent with an isolation by distance model, and has been interpreted as the outcome of basin-scale hydrodynamic processes reducing the mixing of individuals born in different areas of the Adriatic Sea.

Similarly, significant genetic differences were found in this study among populations just a few km far apart (i.e. Lipari-Messina ≈70 Km; Lipari-Ustica ≈150 Km; Ustica-Messina ≈230 Km). Conversely, 5 out of 7 pairwise comparisons between the populations from the Southern Tyrrhenian Sea and the one from the Adriatic Sea (NAD06, separated by a distance of more than 1000 Km from the closest Tyrrhenian sample) did not result statistically significant (P≥0.06934). Moreover, pairwise comparisons among consecutive samples collected in the same locations (Messina, Ustica) in different years showed significant genetic differences, highlighting the lack of temporal stability of the genetic composition of the *P. noctiluca* blooms (Table 7). Altogether these findings suggest the existence of biotic and abiotic mechanisms influencing the genetic pool of *P. noctiluca* to generate a temporally dynamic mosaic of small-scale genetically differentiated patches rather than a homogeneous mixing of the population, or a geographic set of populations isolated by distance.

Genetic heterogeneity on a small geographic scale, especially when temporal genetic differentiation is stronger than spatial differentiation, is generally attributed to temporal changes in the genetic composition of recruits [19], [21], [27]. We suggest that the genetic structure observed in *P. noctiluca* can be explained as fine-scale genetic patchiness, which may be generated through processes driving localised temporal variation of numbers and genotypes of recruits [19]. In such a case, stochastic factors dealing with reproduction processes may influence the proportion of individuals contributing to the next generation, leading to temporal variance in allelic frequencies of the recruits [28]. Under such hypothesis, also called the “hypothesis of sweepstakes reproductive success” (SRS) [28], in species with high fecundity and high mortality rate at early stages, many individuals fail to contribute to recruitment. Several factors as local oceanographic conditions (such as occurrence of canyons and upwelling areas), short life-time of gametes [99] and temporary spatial constraint of individuals can act on fertilization success and formation of recruits pool, generating an “instantaneous drift effect” [27]. Due to the variable parental contribution to recruits pool, the genetic composition of recruits can change generation by generation, leading to spatio-temporal genetic patchiness [100]. From the evolutionary point of view, SRS has important consequences because, due to the stochastic nature of the process involved, divergence is not accumulating but renewed each generation [19], [98], [101] and can be counteracted in the long term by dispersal and gene flow [26], [102]. On the other hand, potentially allowing the replacement of the entire population by a small fraction of individuals, SRS provides the power for rapid evolutionary change and for population resilience [103], [104]. The SRS hypothesis also provides testable predictions [105] such as a reduction of effective population size, measurable effects of genetic drift though time, reduction of allelic diversity and increase of relatedness within cohorts.

To date, many studies provided evidence of a widespread occurrence of chaotic genetic patchiness (CGP) in several benthic marine species [19]–[22], [24]–[26]. However, only very few studies, on fishes [21], [23], [106] and barnacles [31], demonstrated a direct connection among intra population relatedness, family structure and CGP. By considering the high inbreeding and relatedness found at *P. noctiluca* population level, together with the presence of half- and full-siblings in several samples, the present study may contribute to clarify the role of family structure in CGP formation. The most probable scenario to explain the temporally unstable genetic patchiness of *P. noctiluca* populations is the co-occurrence of large variation in the reproductive success of individuals and genetic drift. Indeed, the high intra-population inbreeding level, highlighted by homozygote excess and positive F_IS_ values, combined with the presence of related individuals in several samples could be a symptom of variance in reproductive success generated by environmental, reproductive and behavioural factors. Moreover, the high mortality rate of ephyrae [107]–[109] could generate an instantaneous genetic drift able to enhance the formation of genetically unbalanced assemblage of recruits through random suppression of some allelic variants.

Chaotic genetic variability among populations, explained by alternate periods of rarity and abundance and related genetic drift, is in accordance also with the “Flush and Crash” speciation model [110] as a driving microevolutionary force leading to genetic diversity also at small spatial and temporal scale [111]. *P. noctiluca* populations in the Southern Tyrrhenian Sea are seemingly characterised by a spatio-temporal CGP and random genetic drift may represent a leading micro-evolutionary force shaping the genetic structure of this species. Even if several studies have already shown similar patterns in species with high larval dispersal ability [32], [112]–[116], this study provides the first evidence of family structures and consequent genetic patchiness in a highly dispersive holopelagic species.

## Supporting Information

Table S1 **Loci potentially affected by null alleles.** Results of the Microchecker v. 2.2.3 analysis for each locus and population.
(DOCX)

Table S2 **Hardy Weinberg Equilibrium (HWE) p-values.** HWE p-values were calculated for the original and the corrected dataset (after Microchercker v. 2.2.3 analysis). In bold, significant p-values (α = 0.05). * Results belonging to the dataset corrected with Microchecker v. 2.2.3 are indicated in italic. § For locus Plenoc_16756 no differences are reported due to HWE of all population samples.
(DOCX)

Table S3 **Relatedness Monte Carlo Simulation, null alleles accounted for (adjusted allele frequencies by ML-Relate), 1000 iterations.**
(DOCX)

Table S4 **Relatedness Monte Carlo Simulation, no null alleles accounted for, 1000 iterations.**
(DOCX)

Table S5 **Pairwise FST values calculated using the program FreeNA.** The table S5A shows the results of the analyses performed using the uncorrected dataset. The table S5B is referred to the F_ST_ values obtained implementing the ENA correction method. Pairwise F_ST_ values below the diagonal grey boxes line, lower and upper limits of the 95% confidence interval above the diagonal.
(DOCX)
