# Supplementary material for: First Evidence of Inbreeding, Relatedness and Chaotic Genetic Patchiness in the Holoplanktonic Jellyfish Pelagia noctiluca (Scyphozoa, Cnidaria)
Source: PLoS One. 2014 Jun 30;9(6):e99647. doi: 10.1371/journal.pone.0099647 (PMC4076186; doi:10.1371/journal.pone.0099647)
Supplement: Table S1 — Loci potentially affected by null alleles. Results of the Microchecker v. 2.2.3 analysis for each locus and population. (DOCX) [file pone.0099647.s001.docx]

| **LOCUS** | **Pelnoc** | **Pelnoc** | **Pelnoc** | **Pelnoc** | **Pelnoc** | **Pelnoc** | **Pelnoc** | **Pelnoc** | **Pelnoc** |
| --- | --- | --- | --- | --- | --- | --- | --- | --- | --- |
| **POP** | **40622** | **39456** | **46263** | **44003** | **44210** | **40428** | **40199** | **16756** | **07445** |
| **NAD** | Yes | Yes | Yes | No | Yes | No | Yes | No | Yes |
| **ISC10** | No | No | Yes | Yes | No | No | Yes | No | No |
| **UST10** | No | Yes | Yes | No | Yes | Yes | Yes | No | No |
| **LIP11** | No | Yes | Yes | Yes | Yes | Yes | Yes | No | Yes |
| **MES11** | No | Yes | No | No | No | No | Yes | No | No |
| **UST11** | No | Yes | Yes | Yes | Yes | No | Yes | No | No |
| **MES12** | Yes | Yes | Yes | No | Yes | No | Yes | No | No |
| **UST12** | Yes | Yes | Yes | No | Yes | No | Yes | No | No |
