# Supplementary material for: First Evidence of Inbreeding, Relatedness and Chaotic Genetic Patchiness in the Holoplanktonic Jellyfish Pelagia noctiluca (Scyphozoa, Cnidaria)
Source: PLoS One. 2014 Jun 30;9(6):e99647. doi: 10.1371/journal.pone.0099647 (PMC4076186; doi:10.1371/journal.pone.0099647)
Supplement: Table S2 — Hardy Weinberg Equilibrium (HWE) p-values. HWE p-values were calculated for the original and the corrected dataset (after Microchercker v. 2.2.3 analysis). In bold, significant p-values (α = 0.05). * Results belonging to the dataset corrected with Microchecker v. 2.2.3 are indicated in italic. § For locus Plenoc_16756 no differences are reported due to HWE of all population samples. (DOCX) [file pone.0099647.s002.docx]

| **Locus*** | **NAD** | **UST10** | **UST11** | **UST12** | **ISC10** | **LIP11** | **MES11** | **MES12** |
| --- | --- | --- | --- | --- | --- | --- | --- | --- |
| *Pelnoc_40622* | 0.09950 | 1.00000 | 1.00000 | **0.00064** | 0.23420 | 0.73503 | 1.00000 | **0.01699** |
| Pelnoc_40622 | **0.00180** | 1.00000 | 1.00000 | **P < 0.00001** | 0.23517 | 0.73214 | 1.00000 | **0.00019** |
| *Pelnoc_39456* | **0.00004** | **0.02033** | 0.17934 | **0.00244** | 0.33015 | **0.01980** | 0.27884 | **0.01406** |
| Pelnoc_39456 | **P < 0.00001** | **P < 0.00001** | **P < 0.00001** | **0.00177** | 0.34262 | **P < 0.00001** | **0.02230** | **0.00102** |
| *Pelnoc_46263* | **0.00171** | 0.05351 | 0.29272 | 0.10794 | **0.00718** | **0.01601** | 0.14136 | 0.10344 |
| Pelnoc_46263 | **P < 0.00001** | **P < 0.00001** | 0.16382 | **0.00877** | **0.00926** | **P < 0.00001** | 0.14336 | **0.00745** |
| *Pelnoc_44003* | **0.00352** | **0.00213** | **0.00097** | 1.00000 | 0.41980 | **0.00022** | **0.01680** | **0.02560** |
| Pelnoc_44003 | **0.00432** | **0.00204** | **P < 0.00001** | 1.00000 | **0.02600** | **P < 0.00001** | **0.01706** | **0.02542** |
| *Pelnoc_44210* | 0.48518 | **0.03235** | 0.20777 | 0.48290 | 0.16135 | 0.31604 | 0.56008 | **0.02063** |
| Pelnoc_44210 | **P < 0.00001** | **P < 0.00001** | **0.00012** | **0.01010** | 0.16056 | **0.00039** | 0.55005 | **P < 0.00001** |
| *Pelnoc_40428* | **0.02095** | 0.05605 | 0.07363 | 0.33003 | 0.52319 | 0.32443 | 0.64993 | 0.23928 |
| Pelnoc_40428 | **0.02677** | **0.00119** | 0.07159 | 0.33599 | 0.52089 | **0.02390** | 0.63796 | 0.23860 |
| *Pelnoc_40199* | **0.04260** | **0.00023** | 0.48904 | **0.00837** | 0.06570 | **0.00032** | **0.01479** | **0.00033** |
| Pelnoc_40199 | **P < 0.00001** | **P < 0.00001** | 0.05276 | **0.00195** | 0.06892 | **P < 0.00001** | **0.00210** | **P < 0.00001** |
| Pelnoc_16756 | 0.54782 | 0.20390 | 0.69581 | 0.80848 | 0.65442 | **0.02790** | 0.16851 | 0.80178 |
| *Pelnoc_07445* | **0.03952** | 0.78149 | 0.41268 | **0.01272** | 0.17819 | 0.11025 | 0.91727 | 0.06575 |
| Pelnoc_07445 | **0.00011** | 0.77657 | 0.42206 | **0.01196** | 0.17918 | **0.00022** | 0.92154 | 0.06471 |
