# Supplementary material for: First Evidence of Inbreeding, Relatedness and Chaotic Genetic Patchiness in the Holoplanktonic Jellyfish Pelagia noctiluca (Scyphozoa, Cnidaria)
Source: PLoS One. 2014 Jun 30;9(6):e99647. doi: 10.1371/journal.pone.0099647 (PMC4076186; doi:10.1371/journal.pone.0099647)
Supplement: Table S3 — Relatedness Monte Carlo Simulation, null alleles accounted for (adjusted allele frequencies by ML-Relate), 1000 iterations. (DOCX) [file pone.0099647.s003.docx]

| **Population Pair** | **r_Obs** | **r_Sim_Mean** | **r_Sim_Variance** | **r_Sim_Lower CL** | **r_Sim_Upper CL** | **p-values** |
| --- | --- | --- | --- | --- | --- | --- |
| **Ustica2012Ustica2012** | 0.069756159 | 0.064461986 | 4,13E+00 | 0.077758333 | 0.052442391 | 0.204 |
| **Ustica2011Ustica2012** | 0.062427662 | 0.064602255 | 1,29E+00 | 0.071368519 | 0.057564815 | 0.720 |
| **Ustica2011Ustica2011** | 0.058332222 | 0.064243863 | 1,68E+00 | 0.072904603 | 0.056028730 | 0.936 |
| **Ustica2010Ustica2012** | 0.057180233 | 0.064514385 | 1,04E+00 | 0.071029360 | 0.057993798 | 0.989 |
| **Ustica2010Ustica2011** | 0.069332623 | 0.064378235 | 6,96E-01 | 0.069712597 | 0.059380297 | **0.035** |
| **Ustica2010Ustica2010** | 0.082964009 | 0.064412994 | 1,23E+00 | 0.071591141 | 0.057865338 | **P<0.0001** |
| **Mesina2012Ustica2012** | 0.085417708 | 0.064417572 | 1,95E+00 | 0.072952778 | 0.055956944 | **P<0.0001** |
| **Mesina2012Ustica2011** | 0.060993750 | 0.064263790 | 1,32E+00 | 0.071266667 | 0.057106713 | 0.818 |
| **Mesina2012Ustica2010** | 0.065044380 | 0.064340824 | 1,05E+00 | 0.070424612 | 0.057748256 | 0.433 |
| **Mesina2012Mesina2012** | 0.083258696 | 0.064052093 | 4,13E+00 | 0.076985145 | 0.051075362 | **0.001** |
| **Mesina2011Ustica2012** | 0.051916964 | 0.064440646 | 3,66E+00 | 0.076274702 | 0.053038393 | 0.985 |
| **Mesina2011Ustica2011** | 0.055662103 | 0.064373284 | 2,20E+00 | 0.073512500 | 0.055246627 | 0.972 |
| **Mesina2011Ustica2010** | 0.063789369 | 0.064407276 | 1,91E+00 | 0.073394850 | 0.056545183 | 0.541 |
| **Mesina2011Mesina2012** | 0.058075000 | 0.064595146 | 3,19E+00 | 0.075537202 | 0.053806548 | 0.889 |
| **Mesina2011Mesina2011** | 0.082741758 | 0.064052563 | 0.000123064 | 0.086950549 | 0.043630769 | **0.046** |
| **Lipari2011Ustica2012** | 0.056887343 | 0.064382904 | 9,02E-01 | 0.070419104 | 0.058706053 | 0.992 |
| **Lipari2011Ustica2011** | 0.063015776 | 0.064561373 | 5,72E-01 | 0.069568553 | 0.059929403 | 0.752 |
| **Lipari2011Ustica2010** | 0.066438613 | 0.064454825 | 4,66E-01 | 0.068569460 | 0.060070996 | 0.189 |
| **Lipari2011Mesina2012** | 0.057058726 | 0.064441863 | 8,23E-01 | 0.070122799 | 0.058479717 | 0.995 |
| **Lipari2011Mesina2011** | 0.056397844 | 0.064362685 | 1,57E+00 | 0.072367925 | 0.056491779 | 0.978 |
| **Lipari2011Lipari2011** | 0.062502540 | 0.064410250 | 8,05E-01 | 0.069657402 | 0.058884035 | 0.748 |
| **Ischia2010Ustica2012** | 0.063708013 | 0.064693566 | 3,84E+00 | 0.077009295 | 0.053061538 | 0.555 |
| **Ischia2010Ustica2011** | 0.065642094 | 0.064557310 | 2,44E+00 | 0.074836111 | 0.054964957 | 0.399 |
| **Ischia2010Ustica2010** | 0.067527191 | 0.064187094 | 2,02E+00 | 0.073031306 | 0.055529696 | 0.232 |
| **Ischia2010Mesina2012** | 0.060043590 | 0.064654329 | 4,09E+00 | 0.077495513 | 0.052648077 | 0.761 |
| **Ischia2010Mesina2011** | 0.069837912 | 0.064128226 | 6,58E+00 | 0.081979670 | 0.049352198 | 0.216 |
| **Ischia2010Lipari2011** | 0.072804935 | 0.064320700 | 1,71E+00 | 0.072140929 | 0.056414224 | **0.019** |
| **Ischia2010Ischia2010** | 0.062429487 | 0.064592576 | 0.000146198 | 0.089967949 | 0.042469231 | 0.541 |
