# Supplementary material for: First Evidence of Inbreeding, Relatedness and Chaotic Genetic Patchiness in the Holoplanktonic Jellyfish Pelagia noctiluca (Scyphozoa, Cnidaria)
Source: PLoS One. 2014 Jun 30;9(6):e99647. doi: 10.1371/journal.pone.0099647 (PMC4076186; doi:10.1371/journal.pone.0099647)
Supplement: Table S4 — Relatedness Monte Carlo Simulation, no null alleles accounted for, 1000 iterations. (DOCX) [file pone.0099647.s004.docx]

| **Population Pair** | **r_Obs** | **r_Sim_Mean** | **r_Sim_Variance** | **r_Sim_Lower CL** | **r_Sim_Upper CL** | **p-values** |
| --- | --- | --- | --- | --- | --- | --- |
| **Ustica2012Ustica2012** | 0.067821014 | 0.058737042 | 3,27E+00 | 0.069898188 | 0.048047464 | **0.049** |
| **Ustica2011Ustica2012** | 0.055199653 | 0.05798549 | 1,06E+00 | 0.064580556 | 0.051947454 | 0.800 |
| **Ustica2011Ustica2011** | 0.050952063 | 0.058291291 | 1,47E+00 | 0.06604254 | 0.050944286 | 0.975 |
| **Ustica2010Ustica2012** | 0.050846609 | 0.058285851 | 8,30E-01 | 0.063936143 | 0.052717442 | 0.997 |
| **Ustica2010Ustica2011** | 0.063745930 | 0.058200247 | 5,31E-01 | 0.062626163 | 0.053642765 | **0.009** |
| **Ustica2010Ustica2010** | 0.076477298 | 0.058107915 | 9,79E-01 | 0.064340532 | 0.051789812 | **P<0.0001** |
| **Mesina2012Ustica2012** | 0.078300868 | 0.058456018 | 1,45E+00 | 0.066320486 | 0.051198438 | **P<0.0001** |
| **Mesina2012Ustica2011** | 0.050251620 | 0.058413326 | 1,01E+00 | 0.064796991 | 0.052298727 | 0.996 |
| **Mesina2012Ustica2010** | 0.057425484 | 0.058424115 | 8,76E-01 | 0.064243023 | 0.052594767 | 0.634 |
| **Mesina2012Mesina2012** | 0.073943478 | 0.058291539 | 3,36E+00 | 0.070341304 | 0.047250000 | **0.002** |
| **Mesina2011Ustica2012** | 0.041943155 | 0.058187069 | 2,63E+00 | 0.068795536 | 0.048658631 | 0.999 |
| **Mesina2011Ustica2011** | 0.051936508 | 0.058362330 | 1,85E+00 | 0.066518452 | 0.050141071 | 0.936 |
| **Mesina2011Ustica2010** | 0.060092691 | 0.058223994 | 1,47E+00 | 0.065910133 | 0.050653488 | 0.295 |
| **Mesina2011Mesina2012** | 0.049661012 | 0.058397842 | 2,72E+00 | 0.068272917 | 0.048582143 | 0.953 |
| **Mesina2011Mesina2011** | 0.072723077 | 0.058589130 | 0.000101714 | 0.079192308 | 0.040398901 | **0.049** |
| **Lipari2011Ustica2012** | 0.050779009 | 0.058198727 | 7,12E-01 | 0.063264465 | 0.052986871 | 0.998 |
| **Lipari2011Ustica2011** | 0.058614308 | 0.058361132 | 4,45E-01 | 0.062363155 | 0.054185849 | 0.447 |
| **Lipari2011Ustica2010** | 0.061661694 | 0.058155874 | 3,42E-01 | 0.061857218 | 0.054743747 | **0.036** |
| **Lipari2011Mesina2012** | 0.049918632 | 0.058170257 | 6,72E-01 | 0.063268160 | 0.052996619 | 0.999 |
| **Lipari2011Mesina2011** | 0.051015903 | 0.058321768 | 1,17E+00 | 0.064993531 | 0.051785175 | 0.989 |
| **Lipari2011Lipari2011** | 0.057916328 | 0.058280037 | 5,83E-01 | 0.062919303 | 0.053569739 | 0.556 |
| **Ischia2010Ustica2012** | 0.058112821 | 0.058109820 | 2,98E+00 | 0.069285897 | 0.047634295 | 0.490 |
| **Ischia2010Ustica2011** | 0.061289103 | 0.058226730 | 1,95E+00 | 0.067320299 | 0.049801923 | 0.241 |
| **Ischia2010Ustica2010** | 0.061046691 | 0.058327481 | 1,58E+00 | 0.066134884 | 0.05088068 | 0.254 |
| **Ischia2010Mesina2012** | 0.050874359 | 0.058152237 | 2,86E+00 | 0.069544231 | 0.047809936 | 0.918 |
| **Ischia2010Mesina2011** | 0.058339560 | 0.058073119 | 5,11E+00 | 0.072887912 | 0.045501099 | 0.460 |
| **Ischia2010Lipari2011** | 0.065658345 | 0.057952511 | 1,17E+00 | 0.064574891 | 0.051579971 | 0.016 |
| **Ischia2010Ischia2010** | 0.057485897 | 0.058469924 | 0.000107058 | 0.080289744 | 0.038674359 | 0.527 |
