# Supplementary material for: First Evidence of Inbreeding, Relatedness and Chaotic Genetic Patchiness in the Holoplanktonic Jellyfish Pelagia noctiluca (Scyphozoa, Cnidaria)
Source: PLoS One. 2014 Jun 30;9(6):e99647. doi: 10.1371/journal.pone.0099647 (PMC4076186; doi:10.1371/journal.pone.0099647)
Supplement: Table S5 — Pairwise FST values calculated using the program FreeNA. The table S5A shows the results of the analyses performed using the uncorrected dataset. The table S5B is referred to the FST values obtained implementing the ENA correction method. Pairwise FST values below the diagonal grey boxes line, lower and upper limits of the 95% confidence interval above the diagonal. (DOCX) [file pone.0099647.s005.docx]

| **Table S5A** |  |  |  |  |  |  |  |  |
| --- | --- | --- | --- | --- | --- | --- | --- | --- |
| **POP** | **NAD06** | **UST10** | **ISC10** | **LIP11** | **UST11** | **MES11** | **UST12** | **MES12** |
| **NAD06** |  | 0.00066/0.01306 | -0.01463/0.01556 | -0.00043/0.00540 | -0.00231/0.01092 | -0.004514/0.29236 | 0.00153/0.06879 | -0.00755/0.09371 |
| **UST10** | 0.00597 |  | -0.00942/0.02940 | -0.00268/0.00950 | -0.00337/0.00856 | -0.00017/0.03929 | -0.00335/0.10290 | -0.00159/0.12751 |
| **ISC10** | 0.00074 | 0.00669 |  | -0.01918/-0.12751 | -0.01551/-0.00993 | -0.00797/0.02865 | -0.01205/0.04932 | -0.00297/0.08729 |
| **LIP11** | 0.00276 | 0.00256 | 0.00000 |  | -0.00727/-0.00119 | -0.00514/0.02444 | -0.00575/0.06855 | -0.00165/0.09706 |
| **UST11** | 0.00428 | 0.00246 | 0.00000 | 0.00000 |  | -0.00273/0.02837 | -0.00887/0.05997 | -0.00772/0.08851 |
| **MES11** | 0.01151 | 0.01942 | 0.01122 | 0.00894 | 0.01378 |  | 0.00789/0.10297 | 0.00143/0.12605 |
| **UST12** | 0.02789 | 0.03556 | 0.01146 | 0.01931 | 0.01314 | 0.04653 |  | -0.01740/0.00338 |
| **MES12** | 0.03206 | 0.04153 | 0.03305 | 0.03228 | 0.02574 | 0.04911 | 0.00000 |  |
| **Table S5B** | |  |  |  |  |  |  |  |
| **POP** | **NAD06** | **UST10** | **ISC10** | **LIP11** | **UST11** | **MES11** | **UST12** | **MES12** |
| **NAD06** |  | 0.00241/0.01789 | -0.01067/0.01693 | 0.00162/0.00649 | 0.00041/0.01179 | 0.00188/0.02478 | 0.00253/0.06941 | -0.00481/0.08429 |
| **UST10** | 0.00885 |  | -0.00516/0.02888 | 0.00001/0.00883 | -0.00126/0.00652 | 0.00092/0.03267 | -0.00142/0.12274 | -0.00055/0.13964 |
| **ISC10** | 0.00230 | 0.00981 |  | -0.01117/0.00469 | -0.01214/-0.00009 | -0.00263/0.02274 | -0.01124/0.05854 | 0.00005/0.08367 |
| **LIP11** | 0.00412 | 0.00413 | 0.00000 |  | -0.00577/0.00459 | -0.00373/0.02101 | -0.00198/0.09201 | 0.00159/0.10795 |
| **UST11** | 0.00568 | 0.00240 | 0.00000 | 0.00000 |  | -0.00023/0.02657 | -0.00879/0.08626 | -0.00664/0.10049 |
| **MES11** | 0.01302 | 0.01840 | 0.01006 | 0.00903 | 0.01455 |  | 0.01282/0.12396 | 0.00409/0.13795 |
| **UST12** | 0.02832 | 0.04095 | 0.01605 | 0.03058 | 0.02337 | 0.05334 |  | -0.01198/0.00321 |
| **MES12** | 0.03078 | 0.04690 | 0.03417 | 0.03866 | 0.03069 | 0.05346 | 0.00000 |  |
